# Targeting HIF-1*α* to Prevent Renal Ischemia-Reperfusion Injury: Does It Work?

**DOI:** 10.1155/2018/9852791

**Published:** 2018-11-25

**Authors:** Kapil Sethi, Kenny Rao, Damien Bolton, Oneel Patel, Joseph Ischia

**Affiliations:** ^1^Department of Surgery, Austin Health, University of Melbourne, Heidelberg, VIC, Australia; ^2^Urology Unit, Austin Health, Heidelberg, VIC, Australia

## Abstract

Partial nephrectomy (open or minimally invasive) usually requires temporary renal arterial occlusion to limit intraoperative bleeding and improve access to intrarenal structures. This is a time-critical step due to the critical ischemia period of renal tissue. Prolonged renal ischemia may lead to irreversible nephron damage in the remaining tissue and, ultimately, chronic kidney disease. This is potentiated by the incompletely understood ischemia-reperfusion injury (IRI). A key mechanism in IRI prevention appears to be the upregulation of an intracellular transcription protein, Hypoxia-Inducible Factor (HIF). HIF mediates metabolic adaptation, angiogenesis, erythropoiesis, cell growth, survival, and apoptosis. Upregulating HIF-1*α* via ischemic preconditioning (IPC) or drugs that simulate hypoxia (hypoxia-mimetics) has been investigated as a method to reduce IRI. While many promising chemical agents have been trialed for the prevention of IRI in small animal studies, all have failed in human trials. The aim of this review is to highlight the techniques and drugs that target HIF-1*α* and ameliorate IRI associated with renal ischemia. Developing a technique or drug that could reduce the risk of acute kidney injury associated with renal IRI would have an immediate worldwide impact on multisystem surgeries that would otherwise risk ischemic tissue injury.

## 1. Background: Kidney Cancer and the Limitations of Partial Nephrectomy

There are approximately 338,000 new cases of kidney cancer diagnosed worldwide per year [1]. Partial nephrectomy uses a nephron-sparing approach and is increasingly becoming the gold standard for smaller tumors, tumors in solitary kidneys and patients with underlying poor renal function. Partial nephrectomy usually requires temporary renal arterial occlusion to limit intraoperative bleeding and improve access to intrarenal structures. The two key factors that contribute to postoperative loss of renal function are ischemia time and degree of parenchymal loss [2]. However, while the majority of cancers are suitable for partial nephrectomy, concern about the warm ischemia time has meant that only around 25% of small kidney cancers are treated with nephron-sparing surgery [3]. Current clinical data support a “safe” warm ischemia time of 25 minutes, or a cold ischemia time (when the kidney is placed on ice slush) of 35 min while up to two hours can be tolerated [4]. Beyond this period, critical ischemia ensues, where renal cells are irreversibly injured, eventually resulting in nephron loss and chronic kidney disease in 5-17% patients [4]. Although a large number of drugs and agents (including Ca^2+^-channel blockers, mannitol, acadesine, adenosine, Na^+^/H^+^-exchange inhibitors, and N-acetylcysteine (an antioxidant)) have been shown to protect against ischemia-reperfusion injury (IRI) in the kidney* in vitro* and* in vivo* (in either rat or mouse), all have failed in either large animal or human trials [5–8]. A pharmacological agent that could reduce the risk of IRI and prolong the “safe” warm ischemia time would cause a global transformational change in the utilization of partial nephrectomy, with broader implications for renal transplantation, cardiac surgery, and the myriad other surgeries that involve IRI.

## 2. Mechanisms of I/R Injury

Although the precise pathophysiology of renal IRI is unclear, multiple pathways, which include generation of reactive oxygen species (ROS), apoptosis, hypoxia and associated inducible factors, or inflammation, are involved. In normal cells, the small amount of essential ROS produced during mitochondrial oxidative phosphorylation is neutralized by antioxidant mechanisms. However, during ischemia the activity of the antioxidant enzymes is diminished, leading to an increase in the formation of superoxide radicals and oxidative stress at reperfusion [9]. The free radicals damage proteins, lipids, and DNA, leading to further impairment of mitochondrial function and cell death. During IRI increased apoptosis via activation of a cascade of intracellular proteolytic proteins called caspases has been shown to play a pivotal role in the development of kidney failure [10]. The inflammatory response is another key mechanism which involves endothelial dysfunction and congestion of arterioles by leukocytes and inflammatory cells [11]. Infiltrating leukocytes produce free radicals and cytokines that cause further stromal and epithelial injury.

**Figure 1:**
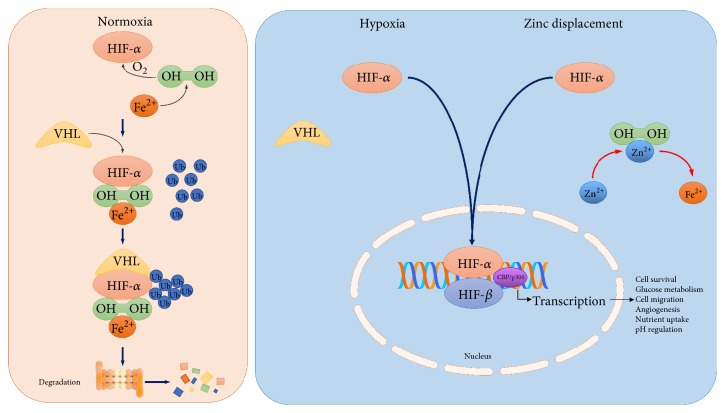
Control of cellular transcription by HIF-*α*. Under normal oxygen tension, HIF-*α* is hydroxylated (OH) through an iron (Fe^2+^) dependent pathway to allow recognition by the von Hippel-Lindau (VHL) gene allowing ubiquitination (Ub) and proteosomal degradation. Hydroxylation cannot occur in hypoxia or displacement of iron by metals such as zinc (Zn^2+^), allowing for HIF-*α* to stabilize and bind with HIF-*β* within the cell nucleus, resulting in the upregulation of cell survival proteins.

One of the major mechanisms of IRI involves pathways downstream of the transcription factor* Hypoxia-Inducible Factor* (HIF). Hypoxia stimulates a protective cellular response characterized by upregulation of a number of specific genes. The key regulator for this process is HIF, a transcription factor that mediates metabolic adaptation, angiogenesis, erythropoiesis, cell growth, survival, and apoptosis [12]. HIF is composed of two subunits: an oxygen-sensitive HIF-*α* subunit, and a constitutively expressed HIF-*β* subunit [13]. At least three isoforms of the *α*-subunit of HIF have been identified. The two most important in the kidney are the widely studied HIF-1*α*, which is predominantly expressed in tubular epithelial cells, and the more recently explored tissue-selective HIF-2*α*, which is almost exclusively found in interstitial, endothelial and fibroblast cells [14]. HIF-1*α* is stabilized by hypoxia, iron chelators or divalent cations. Under normoxic conditions, HIF-1*α* is hydroxylated by a family of iron- and oxygen-dependent enzymes, the prolyl hydroxylases (PHDs) [15]. The hydroxylated form binds to the von Hippel-Lindau tumor suppressor protein (pVHL), as part of a HIF-1*α*/pVHL/ubiquitin ligase complex. Complex formation leads to ubiquitination and proteosomal degradation of HIF-1*α*, and this process keeps HIF-1*α* concentrations low (Figure 1) [16]. Under hypoxic conditions, prolyl hydroxylation is suppressed and the HIF *α*-subunit escapes proteasomal destruction, allowing translocation to the nucleus and dimerisation with HIF-1*β* [17]. Within the nucleus, the dimers of HIF-1*α* or HIF-2*α* with HIF-1*β* regulate the expression of over 100 gene products involved in adaptation for cell survival as a normal physiological response, as well as in oncogenesis [18]. Given this twin role, along with regulation by oxygen tensions, HIF has become an important therapeutic target for manipulation. Drugs may be designed to activate HIF to protect against hypoxia or, equally, HIF-inhibition is a potential antitumor target.

A concern for the oncogenic potential of HIF activators may preclude their use during partial nephrectomy in patients with renal cancer. Reassuringly, early in vivo studies suggest that the carcinogenic potential of such agents remains theoretical without any increase in the rates of cancer. Roxadustat (FG-4592) and daprodustat are PHD inhibitors that have been shown to protect against cisplatin-related kidney injury and upregulate erythropoietin (EPO) in chronic kidney anemia [19, 20]. Sprague Dawley rats exposed to roxadustat showed no greater development of neoplastic lesions at therapeutic dosing for EPO production over two years, and mice bearing a highly aggressive human breast cancer had no evidence of tumor progression [21, 22].

## 3. What Is Preconditioning?

The most effective method of reducing IRI is by cooling the kidney at the time of the operation [23]. However, there is currently no technique or drug that can be given prior to an ischemic insult to prepare the kidney for subsequent ischemia with the goal of ameliorating acute kidney injury in a process called* preconditioning*. Many pharmacological agents such as Ca^2+^-channel blockers, mannitol, adenosine, and N-acetylcysteine have been shown to protect against IRI in preclinical models, but none have proven effective in clinical trials [5–8].

Ischemic preconditioning (IPC) is the innate process of tissue adaptation that results from ischemia or toxic insult, and that subsequently protects against repeated exposure [24]. Preconditioning exploits intracellular signaling pathways to modify gene transcription and enzyme activity, which in turn alter redox reactions, cellular respiration, cellular proliferation and apoptosis, to protect against ischemic injury [25, 26]. Studies have shown that the conditioning stimulus can be effective when applied to either the target organ itself (local ischemic preconditioning; LIPC [27]) or to a remote organ (remote ischemic preconditioning; RIPC [28]).

## 4. Preconditioning Techniques That Target Hypoxic Pathways

### 4.1. Remote Ischemia

Remote ischemia involves causing ischemia, of a limb such as a leg or arm, that when released causes protection of a distant organ such as the heart or kidney. While the exact molecular mechanism of RIPC is not yet fully understood, it is thought to be due to the release of circulating factors such as adenosine, bradykinin, and cannabinoids, which subsequently signal subcellular modulators such as nuclear factor-*κ*B and nitric oxide [24, 29, 30].

In one randomized, single-blind, controlled trial, RIPC protected the kidneys in 120 adult patients undergoing elective cardiac surgery [31]. An automated thigh tourniquet was used to produce three 5-minute intervals of lower extremity ischemia followed by 5 minutes of reperfusion. The authors reported an absolute risk reduction in acute kidney injury of 0.27 and a relative risk reduction of 0.43 [31]. In a recent meta-analysis of animal models of renal injury, IPC caused an overall reduction in peak serum creatinine rise of 54%, in blood urea nitrogen (BUN) levels of 42%, and in histological renal damage (Jablonski score) of 12% [32]. However, it was noted that the degree of study heterogeneity was high.

In a recent well-conducted, randomized clinical trial of RIPC involving three 5-min cycles of right lower limb ischemia and 5 min of reperfusion during each cycle, 82 patients undergoing laparoscopic partial nephrectomy were assessed for changes in renal function at 1 and 6 months using renal scintigraphy [33]. There was a significant difference in the decrease in glomerular filtration rate (GFR) in the affected kidney of 15% in the control group and 8.8% in the RIPC group at 1 month compared to baseline. However, by 6 months, while there was only a 6.1% decrease in GFR in RIPC compared to a 10.5% decrease in control, this difference was not statistically significant [33]. One potential problem with RIPC studies is that they target only the early phase of preconditioning protection. These studies also highlight the difficulties of translating successful animal studies in to humans.

### 4.2. Intermittent Clamping

In 1984, Zager et al. described how prior exposure to kidney ischemia in rats by intermittent clamping (IC) of bilateral renal arteries protected against subsequent ischemic injury [34]. However, IC of the renal artery has only been used in animal models and not extended to any human studies. The prime reason has been the conflicting nature of the published animal results, with no ischemic regimen shown to be significantly superior to others. Heterogeneity of the animal model, clamp time, reperfusion time, number of repeated cycles, critical ischemia time, and follow-up time have proved too many variables to narrow down accurately [35–39]. Most of the animal studies have used 3 or 4 cycles of IC preconditioning similar to that classically applied to the heart [40, 41]. Single episodes of clamping have also been trialed instead of cyclical IC with similar outcomes [42–47]. While many of these studies have shown early promise, a lack of complete mechanistic insight and the confounding nature of the incompletely understood IRI have prevented the important next step of clinical application [48].

### 4.3. Hypoxia-Mimetic Pharmacological Preconditioning of the Kidney

Hypoxia-mimetic agents aim to simulate low intracellular oxygen by inhibiting pathways that are regulated by oxygen tension levels. One mechanism that hypoxia-mimetics target is inhibition of HIF breakdown thereby causing a rise in intracellular HIF (Figure 1).

## 5. Cobalt

Over 60 years ago, the principle of cobalt ions upregulating the production of erythropoietin was commonly applied when cobalt chloride (CoCl_2_) was prescribed for anemia [49]. CoCl_2_ is now recognized as one of the most potent stimulators of HIF [50]. There are several proposed mechanisms for the protective effect of CoCl_2_ in IPC. Cobalt is a transition metal with a 2+ charge which can displace ferrous (Fe^2+^) ions from the active site of PHD enzymes, thereby inhibiting prolyl hydroxylation of HIF (Figure 1) [51]. Iron transport into the cell may also be affected since cobalt ions bind more tightly to a membrane transporter, thereby blocking delivery of ferrous ions into cells [52].

CoCl_2_ has been trialed in various animal models of IRI in recent years. Matsumoto and coworkers demonstrated a profound protective effect on kidneys that underwent 45 minutes of ischemia following CoCl_2_ injection or ingestion [53]. At 48 hours after ischemia, the serum creatinine in the cobalt-preconditioned rats was 42% less than in the control rats. Cobalt-treated rats had substantially reduced tubulointerstitial damage as determined by histological analysis, less macrophage infiltration, and increased mRNA expression of HIF-regulated tissue-protective genes HO-1, EPO, Glut-1, and VEGF [53]. In another study, long-term ingestion of CoCl_2_ for six months in diabetic rats reduced proteinuria and histological kidney damage [54]. Cobalt increased renal expression of HIF-1*α* and HIF-2*α* protein as well as mRNA of the HIF-regulated genes EPO, VEGF, HO-1 [54]. Dosing studies found that saturation of HIF activation occurred at low doses of cobalt with no additional benefit at greater doses that would in any case lead to tissue toxicity [50, 55]. While the above experiments demonstrate proof-of-principle protection against ischemia-reperfusion injury by the transition metal cobalt, it can be quite toxic in animals and therefore has not been investigated further in human trials.

## 6. Zinc

Since the discovery in proteins of the structural element called the “zinc finger” in the early 1980s, recognition of the biological importance of this metallic ion has steadily increased. Zn^2+^ ions play an important role in the physiological functions of all cells, including gene transcription, enzyme activity, cellular proliferation and apoptosis, cellular respiration and redox reactions [25, 26]. For example, Zn^2+^ ions are an essential component of the active site of the enzyme Cu-Zn-superoxide dismutase (SOD) and thus play an essential role in the scavenging of a number of reactive free radicals including superoxide [56]. In addition, Zn^2+^ ions can compete for cellular binding sites with other redox active metal ions such as Fe^2+^, and thus reduce the Fe^2+^-mediated formation of hydroxyl radicals, which are strong oxidants that can cause lipid peroxidation [57].

There have only been four studies that investigated the ability of Zn^2+^ ions to protect against ischemia-reperfusion injury in the kidney. Two decades ago, Hegenauer and coworkers were the first to show that preconditioning with Zn^2+^ ions 30 min prior to one hour of warm ischemia produced a significant improvement in renal function in rabbit kidneys [58]. A more recent study concluded that a single intraperitoneal injection of 20 mg/kg zinc sulfate 24 hours prior to 30 minutes of bilateral renal ischemia ameliorated renal IRI [59]. While GFR was reduced significantly after ischemia, it was significantly better in the zinc-preconditioned animals at 0.76 ml/min compared to 0.41 ml/min in the saline-treated animals. Renal lipid peroxidation, which is a measure of oxidative damage caused by ischemia, was 24% less in the kidneys preconditioned with Zn^2+^ ions [59]. Using a rat model of IRI, we showed that the protective effect of pretreatment with Zn^2+^ ions against renal IRI is dose dependent [60]. We found that preconditioning with subcutaneous 10 mg/kg Zn^2+^ ions 24 and 4 hours prior to 60 minutes ischemia in a single kidney rat model reduced the acute kidney injury by 70%. This effect was not seen at doses of 5 mg/kg or 30 mg/kg [60, 61]. A subsequent study gave mice daily oral Zn^2+^ ions for two weeks prior to 30 minutes of kidney ischemia and showed that zinc ion-preconditioned mice had significantly less histological damage, greater superoxide dismutase activity, and less apoptotic activity [62]. The safety and efficacy of Zn^2+^ ions in renal IRI make it a lead candidate for further investigation.

## 7. Metallothionein

Metallothioneins (MTs) are a family of small cysteine-rich proteins, located in the membrane of the Golgi apparatus in the cells of virtually all living organisms. MTs play an essential role in metal homeostasis, heavy metal detoxification, and as an antioxidant [63]. This is due to the sulfhydryl groups in the cysteine residues in their molecular structure which result in MTs having a high affinity for metal ions, predominantly Zn^2+^ ions at physiological concentrations [64]. In a study of hypoxic rat kidneys after unilateral renal artery stenosis, MT stimulated HIF-1*α* expression [65]. Both hypoxia and MT increased HIF-1*α* in parallel with rises in HIF mRNA, suggesting protein stabilization. The increase in HIF-1*α* appeared to be mediated by signaling through the ERK/mTOR pathway [66].

Metallothionein has also been hypothesized to have antioxidant properties as a result of the redox properties of the cysteine clusters of Zn-MT [67]. Zn-MT can scavenge reactive oxygen species (ROS), by oxidation of its sulfhydryl groups to disulfides. This reaction reduces the metal binding capacity of MT, thereby releasing Zn^2+^ ions to exert their own cellular protective effects. Numerous* in vitro* studies have demonstrated the antioxidant function of MT against ROS and nitrogen species. Du and coworkers used a rat model to investigate the protective effects of Zn-MT against ROS-mediated gentamicin nephrotoxicity* in vitro* and* in vivo* [68]. MT concentrations in the renal cortex of Zn-conditioned rats were significantly higher, and gentamicin-induced proximal tubular necrosis and acute renal failure were ameliorated [68]. Malondialdehyde (MDA) and hydroxyl radical production in the proximal tubules of Zn-treated rats were also significantly lower than those in the control group. A study using MT-overexpressing transgenic mice also added strong evidence for MT protection against apoptosis and IRI in cardiomyocytes [69].

## 8. PHD Inhibitors

Several new agents, that aim to apply the principle of PHD enzyme inhibition for a safe and efficacious effect, show early promise. PHD inhibitors such as the Fe-chelator desferrioxamine (DFO), dimethyloxalylglycine (DMOG), and FG-4487 have demonstrated renoprotection by preconditioning prior to critical ischemia [70–72]. However these agents are less effective than CoCl_2_ in terms of enhancement of HIF-1*α* expression, upregulation of HIF-target gene expression, and conferred protection. The multiple actions of CoCl_2_, rather than iron substitution alone, likely account for its greater benefits. Until our understanding of the protective signaling pathways is further advanced, it is too early to tell whether such PHD inhibitors will be deemed clinically important for renal preconditioning.

## 9. Comparison of Preconditioning Methods

There are very few studies that have directly compared the effects of the different IPC methods, and none in the kidney. A study of porcine hearts found that CoCl_2_ conferred better protection than hypoxia and DFO [73]. In the rat brain, hypoxia was found to be more protective than CoCl_2_ and caused greater upregulation of HIF-1*α* and target genes [74, 75]. A discrepancy has been suggested between the mechanisms of hypoxic and chemical preconditioning, based on differences observed in mRNA and target genes [76]. For example, hypoxia was found to be a greater stimulant of the target gene GLUT-1 than CoCl_2_, which in turn was found to be a stronger activator of the protective protein HO-1 [76–78]. Other important genes are yet to be evaluated, but there is mounting evidence that activation of several cofactor proteins that modulate posttranscriptional regulation may occur simultaneously in target gene regulation [48].

## 10. Conclusion

There are several mechanisms of IRI that lead to acute kidney injury associated with partial nephrectomy (or even renal transplant). Preconditioning drugs (hypoxia-mimetics) and techniques (local or remote ischemic preconditioning) targeting HIF-1*α* have shown great promise in preclinical studies but have all failed in large animal and human trials. It is imperative that future research on IRI explore the role of HIF-1*α*, as well as the other key mechanisms such as apoptosis, generation of reactive oxygen species, inflammation, and cellular stress. The ultimate goal of a drug or technique that could prolong the critical ischemia time would cause a transformational change in the number and complexity of small renal masses that would be suitable for partial nephrectomy and other applications such as transplant and cardiac surgery.
