# Corrigendum to “Targeting HIF-1*α* to Prevent Renal Ischemia-Reperfusion Injury: Does It Work?”

**DOI:** 10.1155/2019/9598038

**Published:** 2019-04-10

**Authors:** Kapil Sethi, Kenny Rao, Arthur Shulkes, Graham Baldwin, Damien Bolton, Oneel Patel, Joseph Ischia

**Affiliations:** ^1^Department of Surgery, Austin Health, University of Melbourne, Heidelberg, VIC, Australia; ^2^Urology Unit, Austin Health, Heidelberg, VIC, Australia

In the article titled “Targeting HIF-1*α* to Prevent Renal Ischemia-Reperfusion Injury: Does It Work?” [1], Drs. Arthur Shulkes and Graham Baldwin were missing from the authors' list who contributed in the formulation of the research goals and the guidance in appropriate areas to focus on the review. In addition, they critically appraised the literature review, edited initial drafts and provided valuable feedback. Finally, they approved the final version of the article. The corrected authors' list is shown above.

## References

[1] Sethi K., Rao K., Bolton D., Patel O., Ischia J. (2018). Targeting HIF-1*α* to prevent renal ischemia-reperfusion injury: does it work?. *International Journal of Cell Biology*.

